# Supplementary material for: Causal Relationship between Aging and Anorexia Nervosa: A White-Matter-Microstructure-Mediated Mendelian Randomization Analysis
Source: Biomedicines. 2024 Aug 16;12(8):1874. doi: 10.3390/biomedicines12081874 (PMC11351342; doi:10.3390/biomedicines12081874)
Supplement: Supplementary file 1 [file biomedicines-12-01874-s001.zip › Supplementary Figure/Figure S7/Single SNP plot of MR analysis for ubm-b-1627 to AN.pdf]

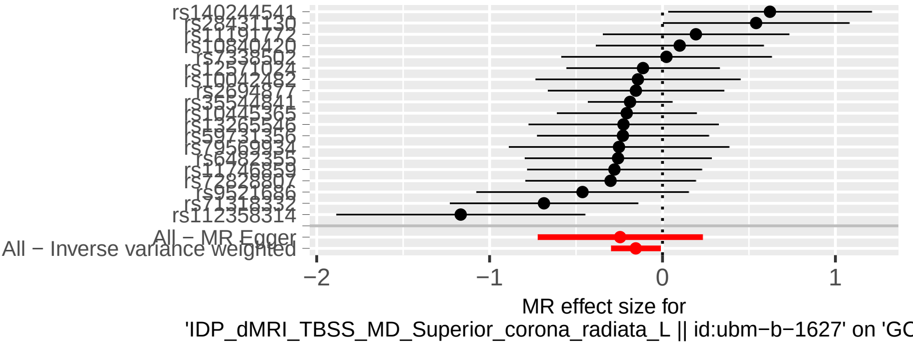
rs140244541
rs28431130
rs11191772
rs10840420
rs7338502
rs12571024
rs10042482
rs2694877
rs35544841
rs10445365
rs13265546
rs59731356
rs79569934
rs6482355
rs11746859
rs72828807
rs9521686
rs71318332
rs112358314
All − MR Egger
All − Inverse variance weighted
−2
−1
0
1
MR effect size for
'IDP_dMRI_TBSS_MD_Superior_corona_radiata_L || id:ubm−b−1627' on 'GC
